# Didymin Suppresses Microglia Pyroptosis and Neuroinflammation Through the Asc/Caspase-1/GSDMD Pathway Following Experimental Intracerebral Hemorrhage

**DOI:** 10.3389/fimmu.2022.810582

**Published:** 2022-01-27

**Authors:** Lingui Gu, Mingjiang Sun, Ruihao Li, Xingyu Zhang, Yihao Tao, Ye Yuan, Xu Luo, Zongyi Xie

**Affiliations:** Department of Neurosurgery, The Second Affiliated Hospital, Chongqing Medical University, Chongqing, China

**Keywords:** intracerebral hemorrhage, Didymin, microglia pyroptosis, neuroinflammation, brain injury

## Abstract

Neuroinflammation has been proven to exert an important effect on brain injury after intracerebral hemorrhage (ICH). Previous studies reported that Didymin possessed anti-inflammatory properties after acute hepatic injury, hyperglycemia-induced endothelial dysfunction, and death. However, the role of Didymin in microglial pyroptosis and neuroinflammation after ICH is unclear. The current study aimed to investigate the effect of Didymin on neuroinflammation mediated by microglial pyroptosis in mouse models of ICH and shed some light on the underlying mechanisms. In this study, we observed that Didymin treatment remarkably improved neurobehavioral performance and decreased BBB disruption and brain water content. Microglial activation and neutrophil infiltration in the peri-hematoma tissue after ICH were strikingly mitigated by Didymin as well. At the molecular level, administration of Didymin significantly unregulated the expression of Rkip and downregulated the expression of pyroptotic molecules and inflammatory cytokines such as Nlrp3 inflammasome, GSDMD, caspase-1, and mature IL-1β, TNF-α, and MPO after ICH. Besides, Didymin treatment decreased the number of Caspase-1-positive microglia and GSDMD-positive microglia after ICH. Inversely, Locostatin, an Rkip-specific inhibitor, significantly abolished the anti-pyroptosis and anti-neuroinflammation effects of Didymin. Moreover, Rkip binding with Asc could interrupt the activation and assembly of the inflammasome. Mechanistically, inhibition of Caspase-1 by VX-765 attenuated brain injury and suppressed microglial pyroptosis and neuroinflammation by downregulation of GSDMD, mature IL-1β, TNF-α, and MPO based on Locostatin-treated ICH. Taken together, Didymin alleviated microglial pyroptosis and neuroinflammation, at least in part through the Asc/Caspase-1/GSDMD pathway *via* upregulating Rkip expression after ICH. Therefore, Didymin may be a potential agent to attenuate neuroinflammation *via* its anti-pyroptosis effect after ICH.

## Introduction

Neuroinflammation has proven to be responsible for the secondary brain injury after intracerebral hemorrhage (ICH), and its mechanisms are characterized by features of microglial activation and neutrophil infiltration to release proinflammatory cytokines, including tumor necrosis factor (TNF)-α, interleukin (IL)-1β, myeloperoxidase (MPO), and other toxic chemicals (1–3). Pyroptosis, a lytic pro-inflammatory type of cell death, is characterized by inflammatory inherently (4, 5). Pyroptosis is strongly associated with the inflammatory response, which features rapid rupture of plasma membrane and release of intracellular contents (4, 6). Furthermore, pyroptosis was also proven to be involved in the pathogenesis of secondary brain injury after ICH (7). Therefore, inhibition of pyroptosis and inflammatory response represents a potential treatment for attenuating secondary brain injury following ICH.

Pyroptosis is induced by the canonical caspase-1 inflammasome complex (8). In concrete terms, activated caspase-1 cleaves gasdermin D (GSDMD), precursors of IL-1β (pro-IL-1β) and IL-18 (pro-IL-18) (9, 10). Gasdermin-N domain fragments are transported to the membrane to build a stable ring-shaped pore (11, 12). The pore is sufficient to enable the passage of inflammatory intracellular contents, triggering cell pyroptosis and consequential excessive inflammation (13, 14). Previous studies have demonstrated that caspase-1 activation and subsequent cleavage of GSDMD exert a crucial effect on pyroptosis and neuroinflammation in central nervous system (CNS) diseases such as cerebral ischemia and Alzheimer’s disease, and inhibiting Caspase-1 activation could mitigate brain injury (15–18). Meanwhile, GSDMD, an executioner of pyroptosis in response to inflammasome activation, which exists in microglia and oligodendrocytes of CNS, has been demonstrated to play a pivotal role in neuroinflammation of multiple sclerosis (MS) and experimental allergic encephalomyelitis (EAE) pathogenesis (19, 20). Thus, modulation of GSDMD-mediated pyroptosis could contribute to attenuating neuroinflammation after ICH.

Didymin, a dietary citrus flavonoid, has been previously reported to possess multiple pharmacological activities including anticancer, antioxidant, anti-inflammation, neuroprotective, hepatoprotective, and cardiovascular activities (21–24). Didymin regulated various important signaling molecules, such as suppressing the MAPK/NF-κB signaling pathway and TLR4/NF-κB and PI3K/Akt pathways, as well as switching M1-like toward M2-like macrophage (21, 23, 25). A previous study showed that Didymin possessed the neuroprotective property of scavenging free radicals and the capability of rescuing the neuronal cells from oxidative damage in neuronal cells after hydrogen peroxide-induced injury (22). In acute liver injury, Didymin could exert an anti-inflammatory effect and alleviate hepatic injury by upregulating the expression of Raf kinase inhibitor protein (RKIP) (23). A recent study reported that Rkip directly bound to apoptosis-associated speck-like protein containing a caspase-recruitment domain (ASC) and interrupted the assembly and activation of the Nlrp3 inflammasome in macrophages (26). However, the effects of Didymin on microglial pyroptosis and neuroinflammation after hemorrhagic stroke have not been elucidated.

In the present study, we hypothesized that Didymin could mitigate microglial pyroptosis and neuroinflammation *via* upregulating Rkip and then suppress the activation of Caspase-1/GSDMD-mediated pyroptosis and neuroinflammation after ICH in mice.

## Materials and Methods

### Animals

A total of 372 C57BL/6 mice (male, weight about 25 g) were purchased from and bred at the Animal Center of Chongqing Medical University. All mice were kept at room temperature (22 ± 1°C) with a 12-h day/night cycle (humidity: 60 ± 5%) with free access to food and water. All procedures involving animals conformed to the Guide for the Care and Use of Laboratory Animals of the National Institutes of Health and were approved by the Institutional Animal Care and Use Committee of Chongqing Medical University.

### Cell Culture

The murine BV2 microglial cell line was obtained from the Shanghai Cell Research Center (Shanghai, China). The cells were cultured in DMEM (4.5 g/l glucose) containing 10% FBS and 1% penicillin/streptomycin at 37°C in a 5% CO_2_ atmosphere. When the cells reach approximately 80% confluence, they were digested with trypsin and passaged for additional experiments. The cells were treated with lipopolysaccharide (LPS (1000 ng/ml) for 4 h. After stimulation, the cells were disrupted using lysis buffer, finally centrifuged, and collected as previously described.

### Experimental Design

**Figure 1:**
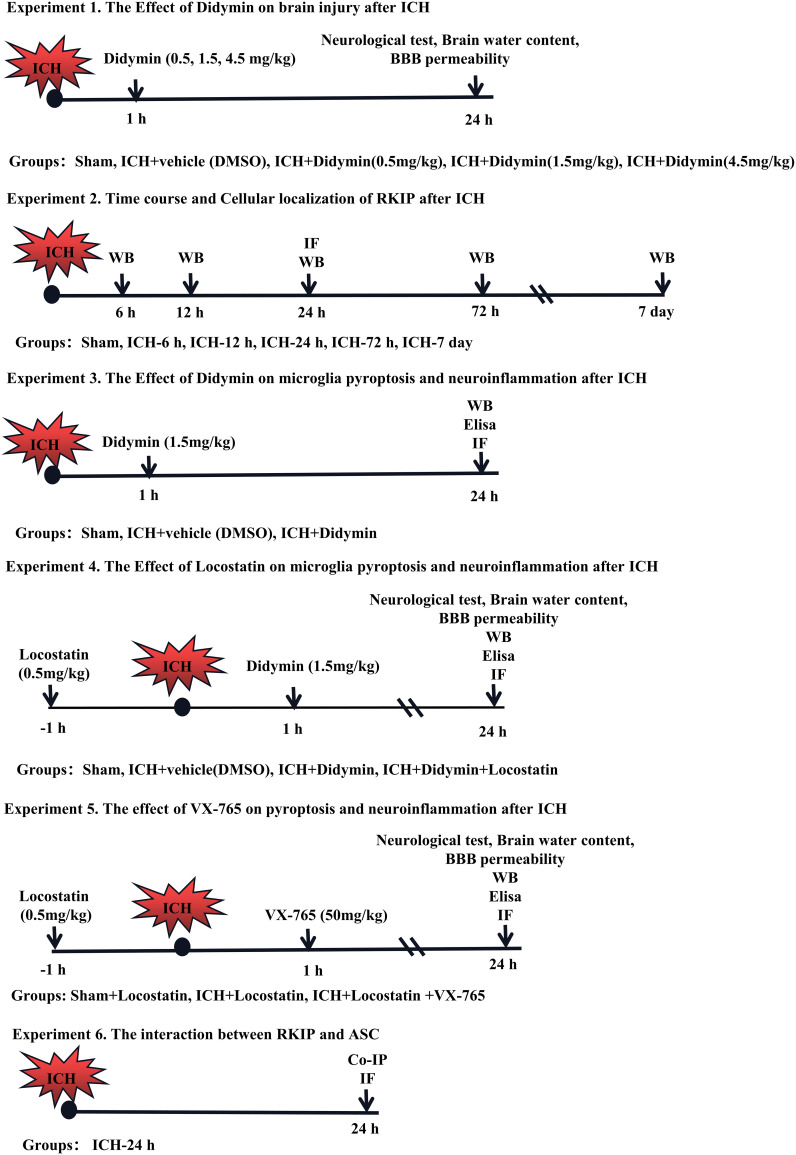
Experimental design and animal groups. Co-IP, co-immunoprecipitation. DMSO, dimethyl sulfoxide. Elisa, enzyme-linked immunosorbent assay. ICH, intracerebral hemorrhage. IF staining, immunofluorescence staining. WB, Western blot.

Six separate experiments were designed as follows (**Figure 1**). A total of 372 mice were used (**Supplementary Table 1**).

**Experiment 1.** The role of Didymin treatment in secondary brain injury after ICH was determined. Three doses of Didymin (0.5, 1.5, 4.5 mg/kg, Solarbio, China) were administered intraperitoneally at 1 h after ICH insult. Mice were randomly divided into 5 groups: sham, ICH+vehicle (DMSO), ICH+Didymin (0.5 mg/kg), ICH+Didymin (1.5 mg/kg), and ICH+Didymin (4.5 mg/kg). Neurological test, brain water content, and Evans blue (EB) extravasation were evaluated at 24 h after ICH.

**Experiment 2.** The temporal expression and cellular localization of Rkip were detected after ICH. Western blot and double immunofluorescence staining were employed in the peri-hematoma tissue at 24 h after ICH.

**Experiment 3.** To evaluate the effects of Didymin on microglial pyroptosis and neuroinflammation after ICH. Western blot, enzyme-linked immunosorbent assay (Elisa), and immunofluorescence staining were performed in the ipsilateral hemisphere after ICH. Mice were randomly divided into 3 groups: sham, ICH+Vehicle (DMSO), and ICH+Didymin (1.5 mg/kg).

**Experiment 4.** To investigate the effect of inhibition of endogenous Rkip on microglial pyroptosis and neuroinflammation, Locostatin (0.5 mg/kg, Darmstadt, Germany), a Rkip-specific inhibitor, was injected intraperitoneally at 1 h before ICH (27), and then Didymin (1.5 mg/kg) was administered intraperitoneally at 1 h after ICH. Neurological test, brain water content and EB extravasation, Western blot, Elisa, and double immunofluorescence staining were performed. Mice were randomly divided into 4 groups: Sham, ICH+Vehicle (DMSO), ICH+Didymin (1.5 mg/kg), and ICH+Didymin (1.5 mg/kg)+Locostatin (0.5 mg/kg).

**Experiment 5.** To explore the effect of inhibition of Caspase-1 on microglial pyroptosis and neuroinflammation, Locostatin (0.5 mg/kg) was injected intraperitoneally in each group at 1 h before ICH insult, and then VX-765 (50 mg/kg, San Diego, CA, USA) (28), a Caspase-1 inhibitor, was administered intraperitoneally at 1 h after ICH insult. Neurological test, brain water content and EB extravasation, Western blot, and Elisa were performed. Mice were randomly divided into 3 groups: Sham+Locostatin (0.5 mg/kg), ICH+Locostatin (0.5 mg/kg), and ICH+Locostatin (0.5 mg/kg)+VX-765 (50 mg/kg).

**Experiment 6.** To investigate the interaction between Rkip and Asc, co-immunoprecipitation (co-IP) of Rkip and Asc was performed. In addition, the co-localization of Rkip and Asc was elucidated by using double-immunofluorescence staining at 24 h after ICH.

### ICH Mouse Model Induction

The ICH model was induced by autologous blood injection as previously described (29). Briefly, the mice were anesthetized with 1% pentobarbital (50 mg/kg, i.p.) and fixed prone in a stereotaxic frame. Next, a small hole about 1 mm in diameter was drilled at 2 mm to the right of the bregma. Then 30 μl autologous arterial blood drawn from the central artery of the tail was delivered into the basal ganglion without anticoagulation (stereotaxic coordinates: 0.2 mm anterior, 2.3 mm right lateral to the bregma, and 3.5 mm ventral to the skull). Finally, 5 μl of blood was injected at 0.7 mm above the target position. The remaining 25 μl of blood was delivered at 3.5 mm depth after the microsyringe was held *in situ* for 5 min. The needle was left for more than 10 min after injection and was withdrawn slowly at a rate of 1 mm/min. Bone wax was then applied to cover the drilled hole. The sham-operated animals were delivered an equal volume of sterile saline at the same position.

### Neurobehavioral Function Test

Neurobehavioral functions were evaluated using the modified Garcia test and corner turn test at 24 h following ICH by a blinded investigator as previously described (30). In the modified Garcia test, seven items including spontaneous activity, axial sensation, vibrissae touch, limb symmetry, lateral turning, forelimb walking, and climbing were tested. Each test was scored as either 0–3 or 1–3, and the total scores ranged from 3 to 21. A higher score indicated a better neurological function. In the corner turn test, mice were allowed to approach a 30° corner. The mice exited the corner with either a right turn or a left turn. Ten trials were performed, with at least a 30-s break between the trials. The percentage of right turn to 10 trials was then calculated.

### Brain Water Content

Brain edema was measured by measuring brain water content at 24 h after ICH *via* an investigator blind to group information as previously described (31). Briefly, mice were decapitated under deep anesthesia. The brain was immediately removed and cut into a 4-mm coronal slice and separated into five parts: ipsilateral and contralateral basal ganglia, ipsilateral and contralateral cortex, and cerebellum. The cerebellum was retained as an internal control. Each part was immediately weighed on an electronic analytical balance (FA2204B, Techcomp, USA) to obtain the wet weight (WW) and then dried at 100°C for 72 h to obtain the dry weight (DW). Brain water content (%) was calculated using the following formula: brain water content (%) = [(wet weight − dry weight)/wet weight] × 100%.

### BBB Permeability

To evaluate BBB permeability, Evans blue (Aladdin, China) was injected intraperitoneally (100 μl of 4% solution in saline) as previously described with a slight modification (32). After 3 h of circulation, mice were transcardially perfused with cold phosphate-buffered saline (0.1 M, PBS, pH 7.4) under deep anesthesia. Afterward, the brain was removed and divided into the left and right hemispheres, stored at -80°C immediately. The right part of the brain was homogenized in 1,100 μl PBS, sonicated, and centrifuged (12,000 g, 4°C, 30 min). The supernatant was collected, and an equal amount of trichloroacetic acid (TCA) was added and incubated overnight by 4°C. After centrifugation (12,000 g, 4°C, 30 min), Evans blue stain was measured by a spectrophotometer (Thermo Fisher Scientific, USA) at 610 nm.

### Immunofluorescence Staining

Double fluorescence staining was performed as described previously (33). The mice were deeply anesthetized and were transcardially perfused with 20 ml ice-cold PBS followed by 20 ml of 4% paraformaldehyde at 24 h post-ICH. The whole brain was collected and then fixed in 4% paraformaldehyde for another 24 h. Afterward, the brain was dehydrated in 20% sucrose solution and 30% sucrose solution. After being frozen at −25°C, the brain was cut into 10-μm-thick coronal sections using a cryostat (CM1860; Leica Microsystems, Germany). To conduct double immunohistochemistry staining, the brain sections were incubated at 4°C overnight with a primary antibody: anti-Transmembrane protein (TMEM119, 1:100, Proteintech, China), anti-glial fibrillary acidic protein (GFAP, 1:200, CST, USA), anti-Neun (1:100, Abcam, USA), anti-MPO (1:200, Santa Cruz, USA), and anti-caspase-1 p20 (1:100, Abcam, USA) and anti-GSDMD (1:500, Santa Cruz, USA), After being incubated with the appropriate secondary antibody (1:200, Bioss, China) at 37°C for 1 h, the sections were visualized and photographed with a fluorescence microscope (U-HGLGPS, OLYMPUS, Japan). Microphotographs were analyzed with cellSens Standard software.

The LPS-treated BV2 cells were seeded on glass coverslips in 24-well plates. After various treatments, the cells were fixed in 4% paraformaldehyde at room temperature for 20 min and then washed thrice in PBST. After permeabilization with 0.1% Triton X-100/PBS for 15 min, the cells were washed with PBS, blocked in PBS with 5% BSA at room temperature for 1 h, and then incubated with RKIP (1:100, Abcam, USA) and ASC (1:50, Santa Cruz, USA) primary antibodies at 4°C overnight. After being washed with PBS, the cells were incubated with FITC-conjugated secondary antibodies (1:1,000) for 1 h at room temperature. Finally, the cells were washed with PBS, mounted in Fluoroshield containing DAPI, and analyzed by confocal microscopy

### Western Blot

Western Blotting was performed as previously described (34). Briefly, after mice were perfused with ice-cold PBS (0.1 M, pH 7.4) at 24 h postoperation, the peri-hematoma tissues were collected and stored in a -80°C freezer until use. After brain protein sample preparation using RIPA lysis buffer (Santa Cruz Biotechnology, Santa Cruz, CA, USA), equal amounts of protein were loaded onto an SDS-PAGE gel and run using electrophoresis. After being electrophoresed and transferred to a PVDF membrane, the membrane was blocked for 2 h at 37°C followed by incubation with the primary antibody overnight at 4°C. The primary antibodies were anti-RKIP (1:1,000, Abcam, USA), anti-ASC (1:1,000, Abcam, USA), anti-NLRP3 (1:1,000, Abcam, USA), anti-GSDMD (1:1,000, Abcam, USA), anti-caspase-1 (1:1,000, Abcam, USA), and anti-β-actin (1:5,000, Proteintech, China). The secondary antibodies (ZSGB-BIO) were incubated for 1 h at room temperature. The bands were probed with an ECL Plus Chemiluminescence Reagent Kit (Amersham Biosciences, Arlington Heights, PA, USA) and visualized with the image system (VersaDoc, model 4000, Bio-Rad, Hercules, CA, USA). The relative density of the protein immunoblot images were analyzed by ImageJ software (ImageJ 1.4, NIH, Bethesda, MD, USA).

### Co-Immunoprecipitation

Co-immunoprecipitation was performed as previously described (35). Tissue protein lysates were obtained with NP40 buffer (150 mM NaCl, 50 mM Tris–HCl [pH 7.5], 1% NP40) containing a protease and phosphatase inhibitor cocktail (Thermo Fisher Scientific). 60 μl tissue protein was taken as the input group. Protein G-agarose beads (Thermo) were washed 3 times with washing buffer [50 mm Tris (pH 7.5), 0.1 mm ethylenediaminetetraacetic acid, 1% Triton X-100, 10% glycerol, 1 mm phenylmethylsulfonyl fluoride, and 1 mm DTT]. Protein G-agarose (Thermo) beads were added with capturing antibody for 2 h at 4°C and then washed with the washing buffer. For immunoprecipitation, 1 mg of total protein was used with 40 μl of Protein G Dynabeads precoated with 2 μl of capturing antibody. Tissue proteins were incubated with the mixed sample. After overnight incubation, beads were extensively washed 5 times with NP40 buffer. Samples were eluted by incubation with 1× concentrated electrophoresis sample buffer and 2× concentrated electrophoresis sample buffer added in the input group, then both were boiled for 10 min. The supernatant was collected to proceed to western blot.

### Enzyme-Linked Immunosorbent Assay

Enzyme-linked immunosorbent assay (ELISA) was performed as previously described (36). Tissue protein from experimental animals with ICH was obtained and detected by ELISA kit (R&D Systems, USA), according to the manufacturer’s instruction. At 24 h after ICH, mice were anesthetized and then perfused with 0.1 M PBS. Tissue protein from experimental animals with ICH was obtained and homogenized in lysis buffer containing protease inhibitors (to each 10 mg of tissue, 100 μl of lysis buffer was used); the supernatant without dilution was used to measure IL-1β, TNF-α, and MPO, respectively. A Mouse DuoSet ELISA Kit to IL-1β, TNF-α, and MPO was purchased from R&D Systems and performed as instructed by the manufacturer. The protein concentration of the lysate was determined using a Pierce™ bicinchoninic acid assay protein assay kit (Thermo Scientific, Rockford, IL, USA).

### Statistics Analysis

All data were expressed as mean and standard deviation (mean ± SD). All analyses were performed using SigmaPlot 11.0 and GraphPad Prism 8 (GraphPad Software, San Diego, CA). Firstly, the Shapiro–Wilk normality test was implemented in determining data normality. For the data that conformed to a normal distribution, one-way ANOVA analysis followed by Tukey *post hoc* test was used for multiple-group comparisons. For the data that failed the normality test, Kruskal–Wallis one-way ANOVA on ranks, followed by Tukey multiple-comparison *post hoc* analysis, was performed. Statistical differences between the two groups were analyzed using the Student’s unpaired, two-tailed t-test. A p-value less than 0.05 was defined as statistically significant.

## Results

### Mortality and Exclusion

The total mortality of ICH mice was 8.06% (30/372) in this study. None of the sham group mice died. There was no significant difference in mortality rate among experimental groups. 8 mice were ruled out from this study due to no hematoma **(** **Supplementary Table 1**).

### Didymin Treatment Attenuated Neurobehavioral Deficits, BBB Permeability, Brain Edema, and Hematoma Size After ICH

**Figure 2:**
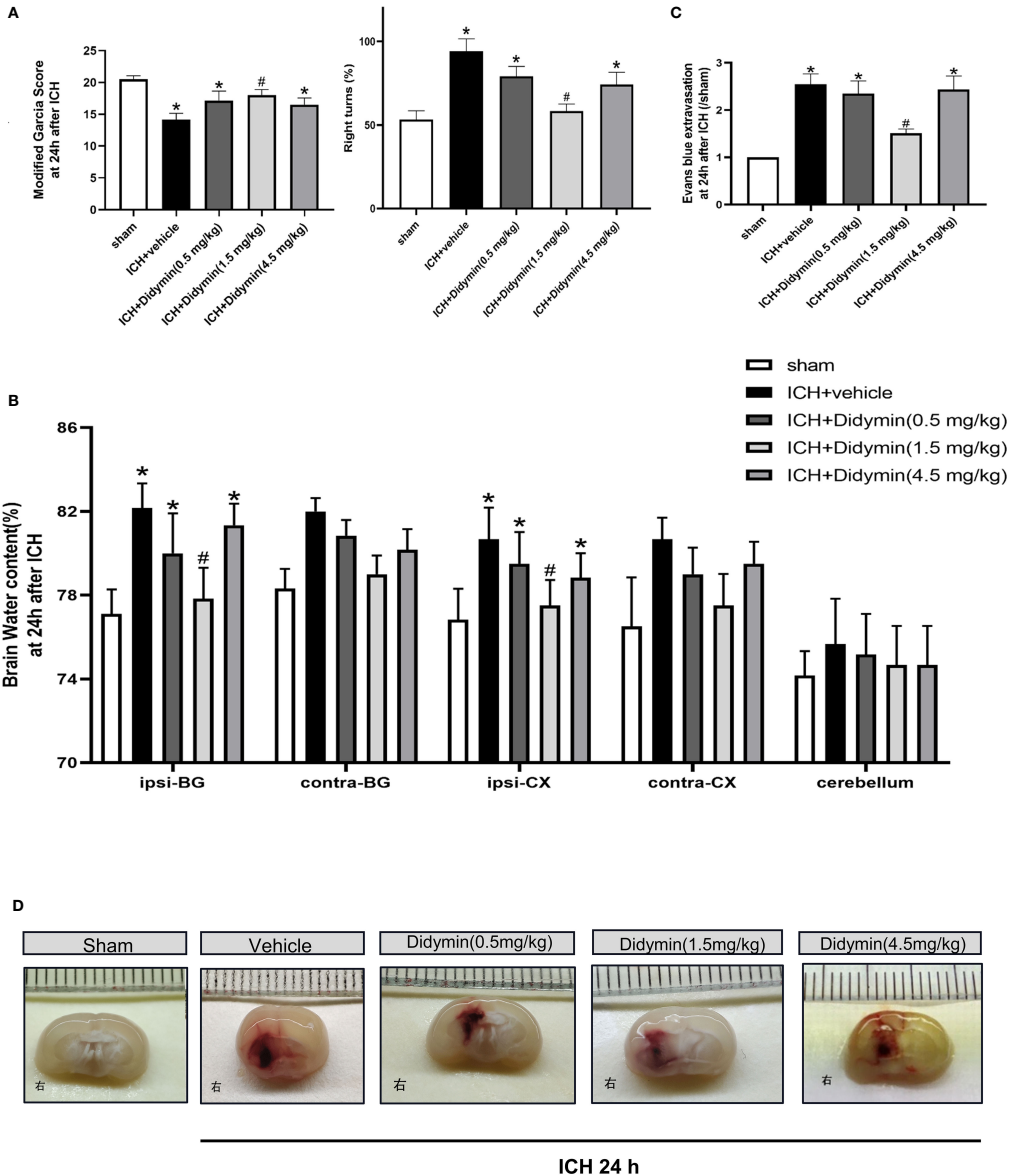
Neuroprotective effects of Didymin on brain injury after ICH. **(A–D)** Neurological deficits, brain water content, EB extravasation, and hematoma size were tested at 24 h after ICH. Ipsilateral basal ganglia (ipsi-BG), contralateral basal ganglia (contra-BG), ipsilateral cortex (ipsi-CX), contralateral cortex (contra-CX), and cerebellum. n = 6 per group. ^*^ *p* < 0.05 vs. sham; ^#^ *p* < 0.05 vs. ICH+vehicle.

The neurological deficits, brain edema, and BBB permeability assessed by EB extravasation were worse in the ICH+Vehicle group, ICH+Didymin (0.5 mg/kg) group, and ICH+Didymin (4.5 mg/kg) group, when compared with the sham group at 24 h after ICH (**Figures 2A, B**). Administration of Didymin (1.5 mg/kg) markedly improved the neurological impairments (**Figure 2A**), reduced brain edema (**Figure 2B**), alleviated the BBB permeability (**Figure 2C**) and hematoma size (**Figure 2D**) compared with those in the ICH+Vehicle group. Based on these results, the optimal dose of Didymin was 1.5 mg/kg, which was set as a standard dose for the following studies.

### Expression of Endogenous Rkip in the Peri-Hematoma Tissue After ICH

**Figure 3:**
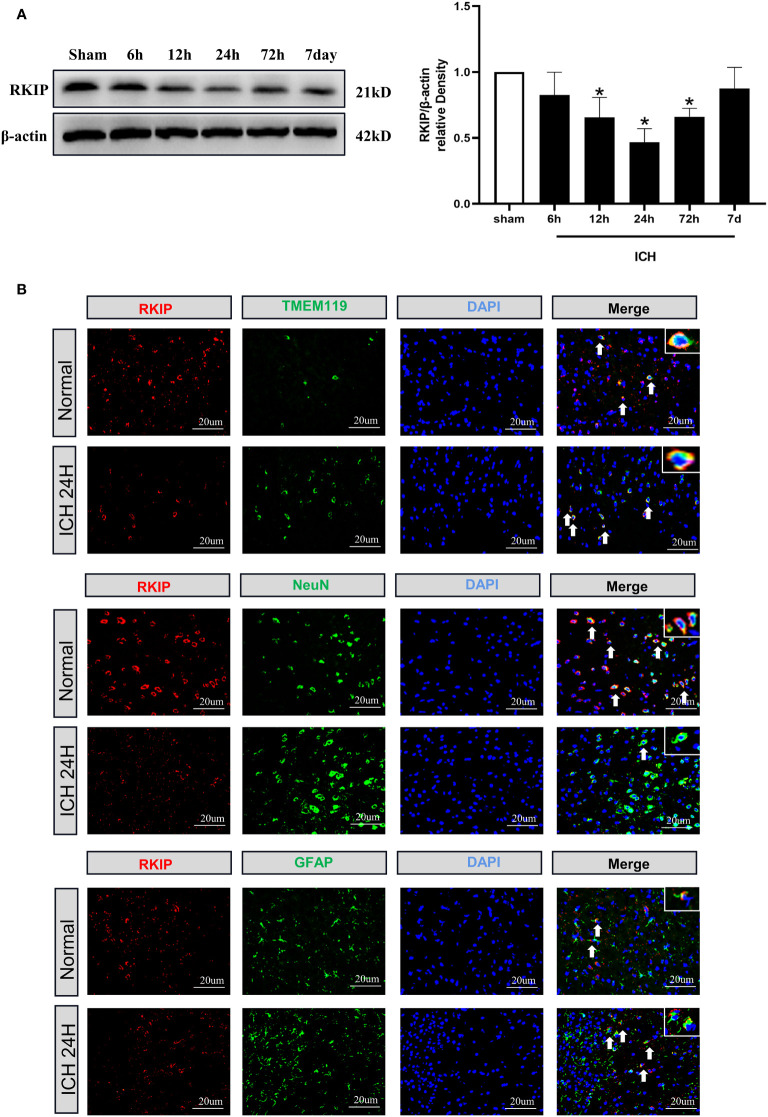
The temporal expression and cellular localization of Rkip after ICH. **(A)** Representative Western blot band and quantitative analyses of Rkip time-dependent expression from the peri-hematoma tissue after ICH. n = 6 per group. *p < 0.05 vs. sham. **(B)** Representative images of double immunofluorescence staining showed that Rkip was co-localized with microglia (TMEM119), astrocytes (GFAP), and neurons (NeuN) separately at 24 h after ICH. Scale bar = 20 μm. n = 3 per group.

Western blot analysis showed that endogenous Rkip expression in the peri-hematoma tissue was significantly decreased at 24 h after ICH compared with the sham group (**Figure 3A**). Double immunofluorescence staining showed that Rkip was mainly expressed in microglia, astrocytes, and neurons in the peri-hematoma tissue at 24 h after ICH (**Figure 3B**).

### Didymin Treatment Suppressed Microglial Activation and Neutrophil Infiltration After ICH

**Figure 4:**
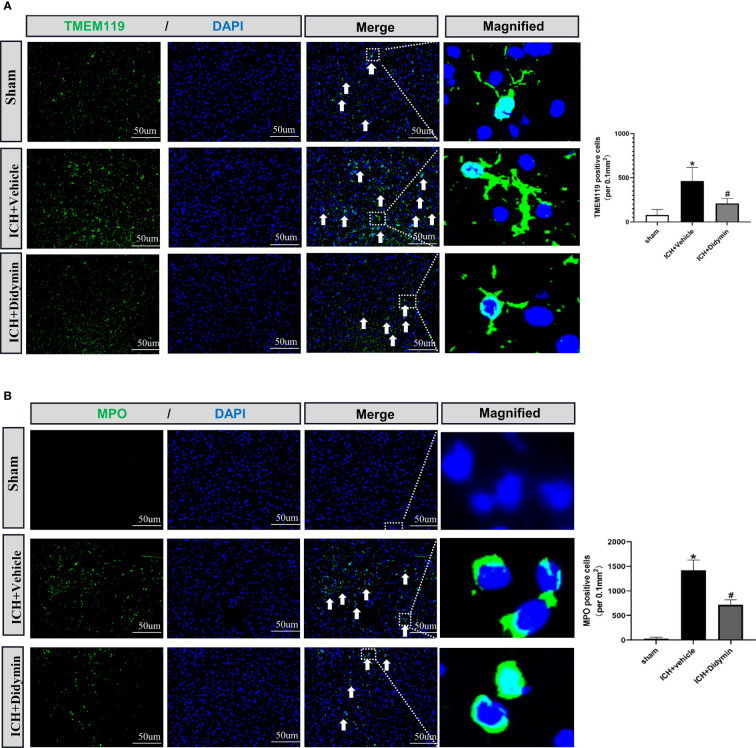
Didymin treatment suppressed microglial activation and neutrophil infiltration. **(A, B)** Images of TMEM119 and MPO immunostaining and quantitative analysis of Iba-1-positive and MPO-positive cells. Scale bar = 50 μm. n = 3 for each group. ^*^ *p* < 0.05 vs. sham; ^#^ *p* < 0.05 vs. ICH+vehicle.

To investigate the effect of Didymin on neuroinflammation, we visualized activated microglia using Iba-1 staining and neutrophil infiltration by MPO staining. As depicted in **Figure 4A**, the number of TMEM119-positive cells was markedly increased in the peri-hematoma tissue at 24 h after ICH, while treatment with Didymin diminished the number of TMEM119-positive cells. MPO staining results showed that ICH induced a significant increase of MPO-positive cells in the ipsilateral hemisphere, while Didymin treatment significantly reversed the trend (**Figure 4B**).

### Didymin Ameliorated Microglial Pyroptotic Molecules and Inflammatory Cytokines After ICH

**Figure 5:**
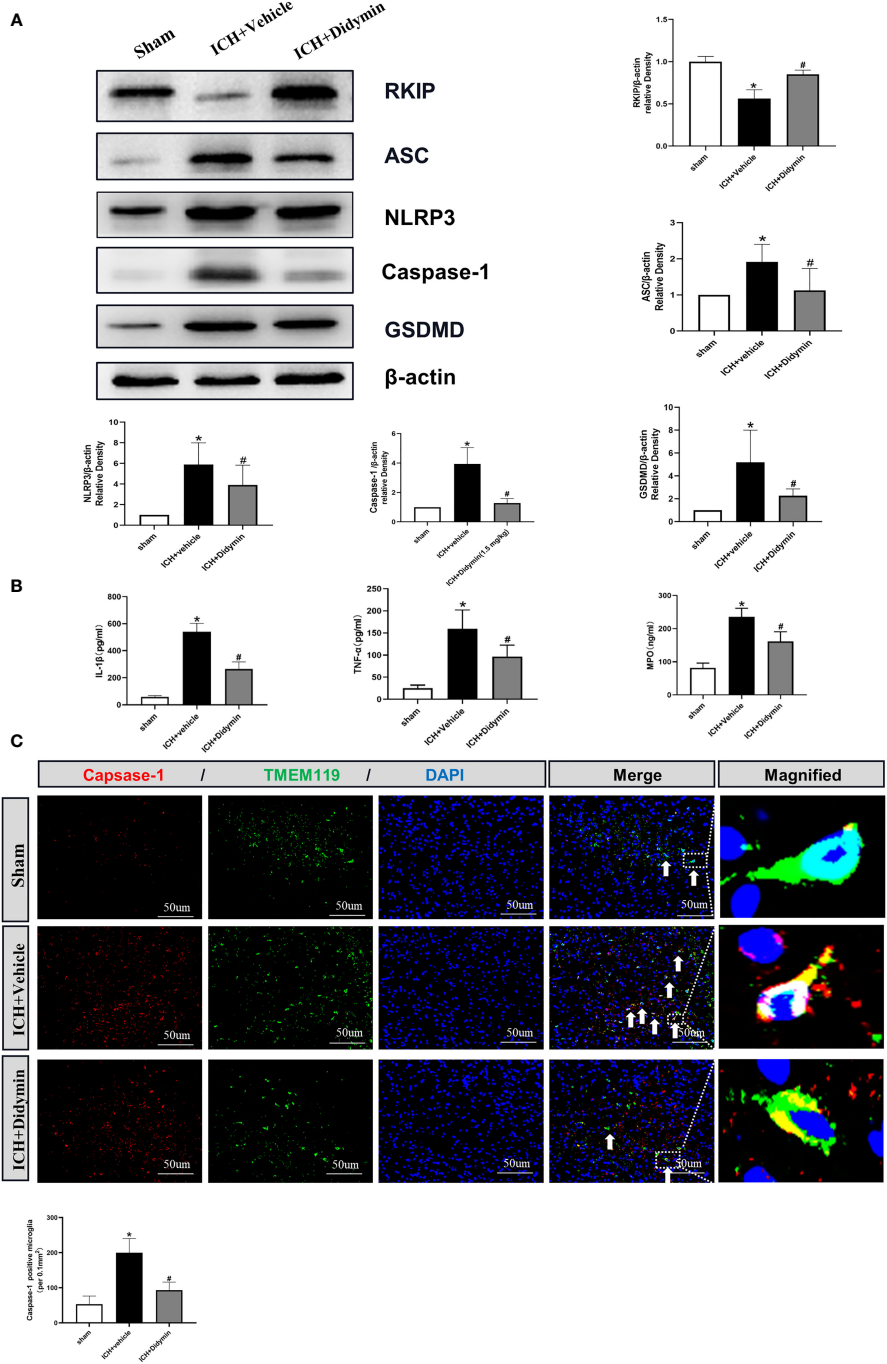
Didymin ameliorated microglial pyroptotic molecules and inflammatory cytokines after ICH. **(A)** Representative Western blot bands and densitometric quantification of Rkip, Asc, Nlrp3, Caspase-1, and GSDMD. n = 6 for each group. ^*^ *p* < 0.05 vs. sham; ^#^ *p* < 0.05 vs. ICH+vehicle. **(B)** IL-1β, TNF-α, and MPO production were detected by ELISA assay kit. n = 6 for each group. ^*^ *p* < 0.05 vs. sham; ^#^ *p* < 0.05 vs. ICH+vehicle. **(C)** Caspase-1/TMEM119 double immunofluorescence staining and quantitative analysis of Caspase-1-positive microglia. Scale bar = 50 μm. n = 3 for each group. ^*^ *p* < 0.05 vs. sham; ^#^ *p* < 0.05 vs. ICH+vehicle.

Western blot analysis showed that Rkip expression was decreased after ICH, accompanied by the upregulation of pyroptotic molecules such as Asc, Nlrp3, Caspase-1, and GSDMD (**Figure 5A**). However, Didymin treatment upregulated Rkip expression and downregulated the expression of pyroptotic molecules (**Figure 5A**). ELISA results showed that Didymin treatment decreased inflammatory cytokines such as mature IL-1β, TNF-α, and MPO at 24 h after ICH (**Figure 5B**). Immunofluorescence staining results showed that the amount of Caspase-1-positive microglia in the peri-hematoma tissue was increased at 24 h after ICH. However, Didymin treatment decreased the amount of Caspase-1-positive microglia in the peri-hematoma tissue at 24 h after ICH (**Figure 5C**).

### Inhibition of Endogenous Rkip Abolished the Neuroprotective Effects of Didymin After ICH

**Figure 6:**
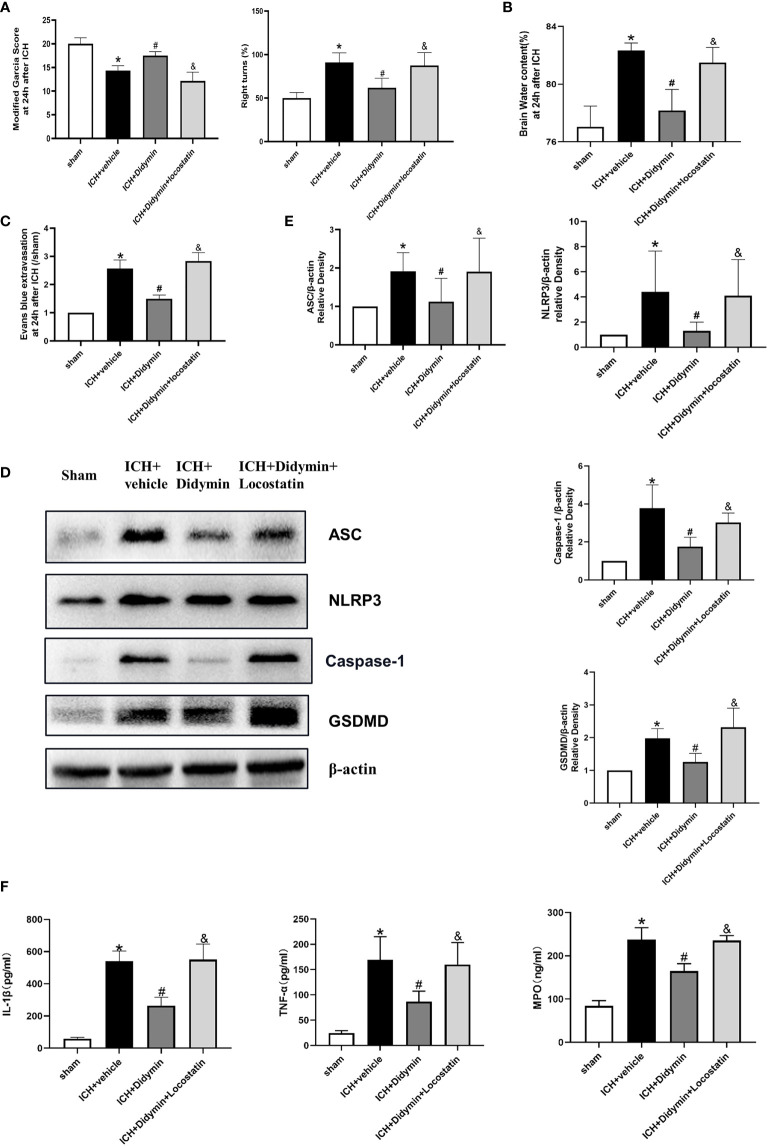
Locostatin abolished the neuroprotective effects of Didymin. **(A–C)** Neurological deficits, brain water content, and EB extravasation were tested at 24 h after ICH. **(D, E)** Representative Western blot bands and densitometric quantification of Asc, Nlrp3, Caspase-1, and GSDMD. n = 6 for each group. *p < 0.05 vs. sham; ^#^ *p* < 0.05 vs. ICH+vehicle. ^&^ *p* < 0.05 vs. ICH+Didymin. **(F)** IL-1β, TNF-α, and MPO production was detected by ELISA assay kit. n = 6 for each group. *p < 0.05 vs.sham; ^#^ *p* < 0.05 vs. ICH+vehicle. ^&^ *p* < 0.05 vs. ICH+Didymin.

To explore whether the neuroprotective effects of Didymin are related to Rkip, Locostatin, an Rkip-specific inhibitor, was injected at 1 h before ICH insult, and then Didymin was injected at 1 h after ICH. Inhibition of endogenous Rkip significantly aggravated neurological deficits (**Figure 6A**), brain edema (**Figure 6B**), and EB extravasation (**Figure 6C**) in ipsilateral basal ganglion at 24 h after ICH compared with the ICH+Didymin group. Western blot and ELISA results showed that Locostatin increased the expressions of Asc, Nlrp3, Caspase-1, GSDMD, mature IL-1β, TNF-α, and MPO compared with the ICH+Didymin group (**Figures 6D–F**).

### Inhibition of Caspase-1 Alleviated Microglial Pyroptosis and Neuroinflammation After Locostatin-Treated ICH

**Figure 7:**
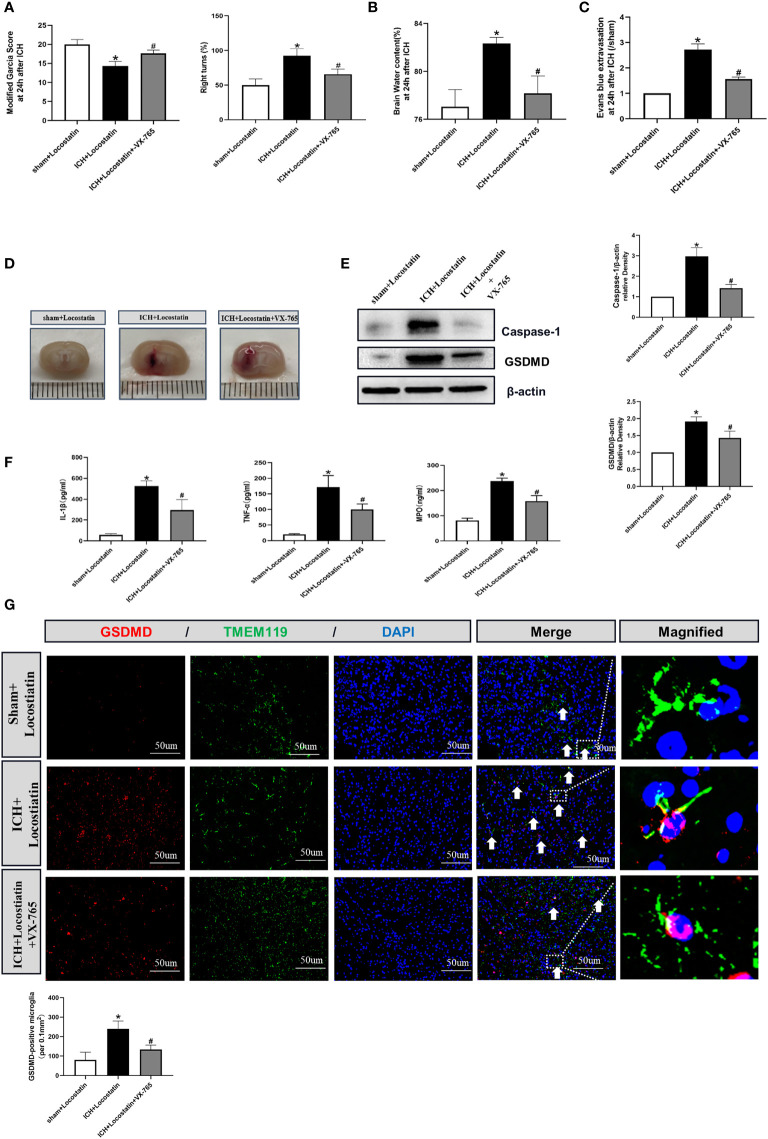
VX-765 ameliorated microglial pyroptosis and brain injury after Locostatin-treated ICH. **(A–D)** Neurological deficits, brain water content, EB extravasation, and hematoma size were tested at 24 h after ICH. **(E)** Representative Western blot bands and densitometric quantification of Asc, Nlrp3, Caspase-1, and GSDMD. n = 6 for each group. ^*^ *p* < 0.05 vs. sham; ^#^ *p* < 0.05 vs. ICH+vehicle. **(F)** IL-1β, TNF-α, and MPO production was tested by ELISA assay. n = 6 for each group. ^*^ *p* < 0.05 vs. sham; ^#^ *p* < 0.05 vs. ICH+vehicle. **(G)** GSDMD/TMEM119 double immunofluorescence staining and quantitative analysis of GSDMD-positive microglia. Scale bar = 50 μm. n = 3 for each group. ^*^ *p* < 0.05 vs. sham; ^#^ *p* < 0.05 vs. ICH+vehicle.

To further confirm whether the protective effect of Didymin on microglial pyroptosis and neuroinflammation is mediated by the Caspase-1/GSDMD pathway, we adopted the reversed verification method. VX-765, a Caspase-1 selective inhibitor, was injected based on Locostatin-treated ICH. As shown in **Figures 7A–D**, VX-765 alleviated neurological deficits, brain edema, EB extravasation, and hematoma size in the ICH+Locostatin+VX-765 group compared with the ICH+Locostatin group. Western blot and ELISA results showed that the expression of Caspase-1, GSDMD, mature IL-1β, TNF-α, and MPO was decreased in the ICH+Locostatin+VX-765 group compared with the ICH+Locostatin group (**Figures 7E, F**). Similar results were observed by the immunofluorescence staining assay. The number of GSDMD-positive microglia in the peri-hematoma tissue was decreased at 24 h after ICH in the ICH+Locostatin+VX-765 group compared with the ICH+Locostatin group (**Figure 7G** **).**

### Interaction of Rkip With Asc

**Figure 8:**
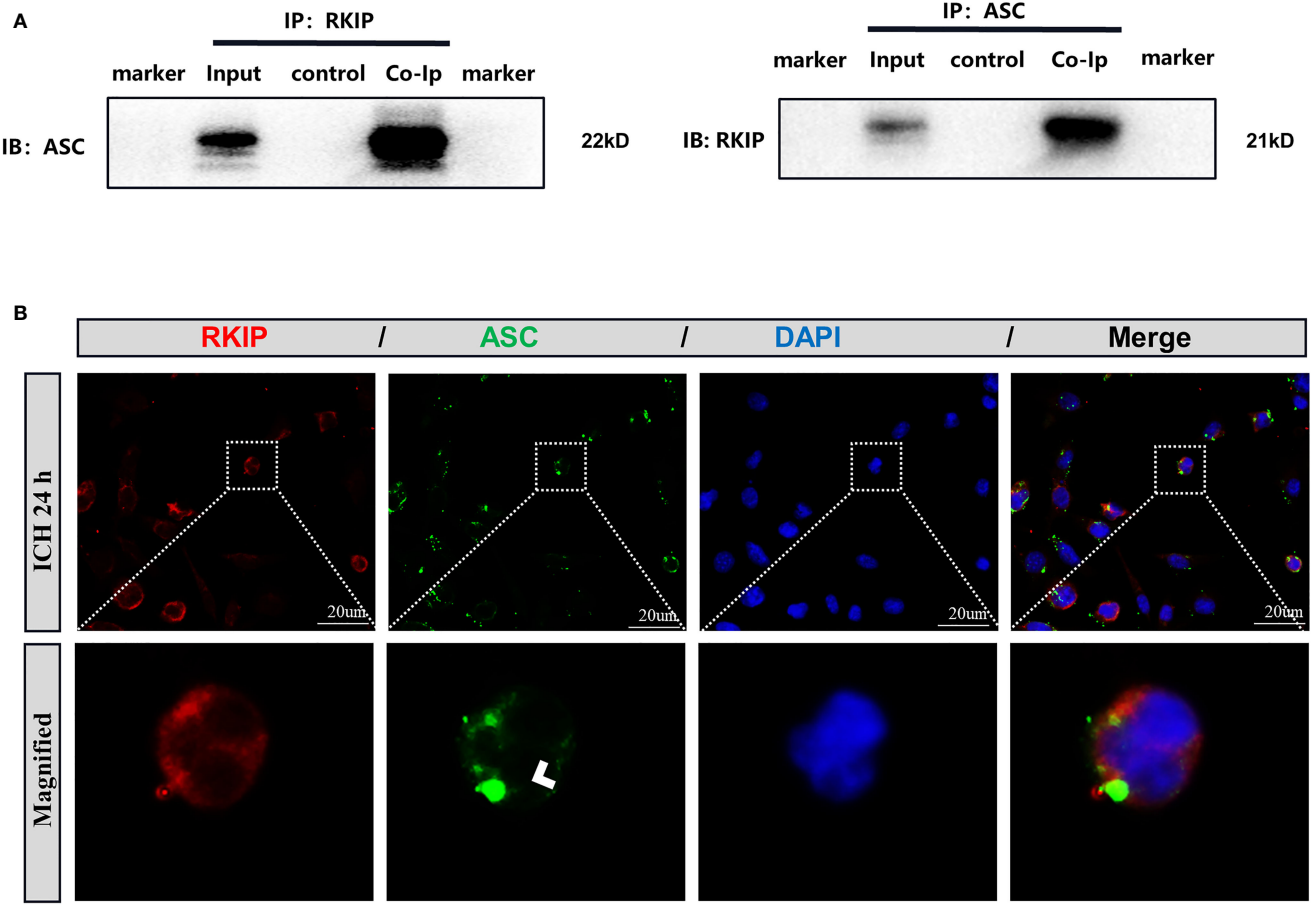
Rkip directly interacts with Asc. **(A)** Co-immunoprecipitation of Rkip and Asc n = 6 for each group. **(B)** Rkip/Asc double immunofluorescence staining showed that Rkip combined with Asc co-expressed after LPS stimulation in the BV-2 cells. Scale bar = 20 μm. n = 3 for each group.

To further study the mechanism of Didymin-mediated pyroptosis after ICH, co-immunoprecipitation was performed. Results showed that Rkip was related to Asc (**Figure 8A**). Co-immunostaining result showed that both Rkip and Asc expressed in the cytoplasm after LPS stimulation in BV-2 cells (**Figure 8B**). These results indicated that Didymin upregulated Rkip, which directly bound Asc to interrupt Nlrp3 inflammasome formation after ICH.

## Discussion

**Figure 9:**
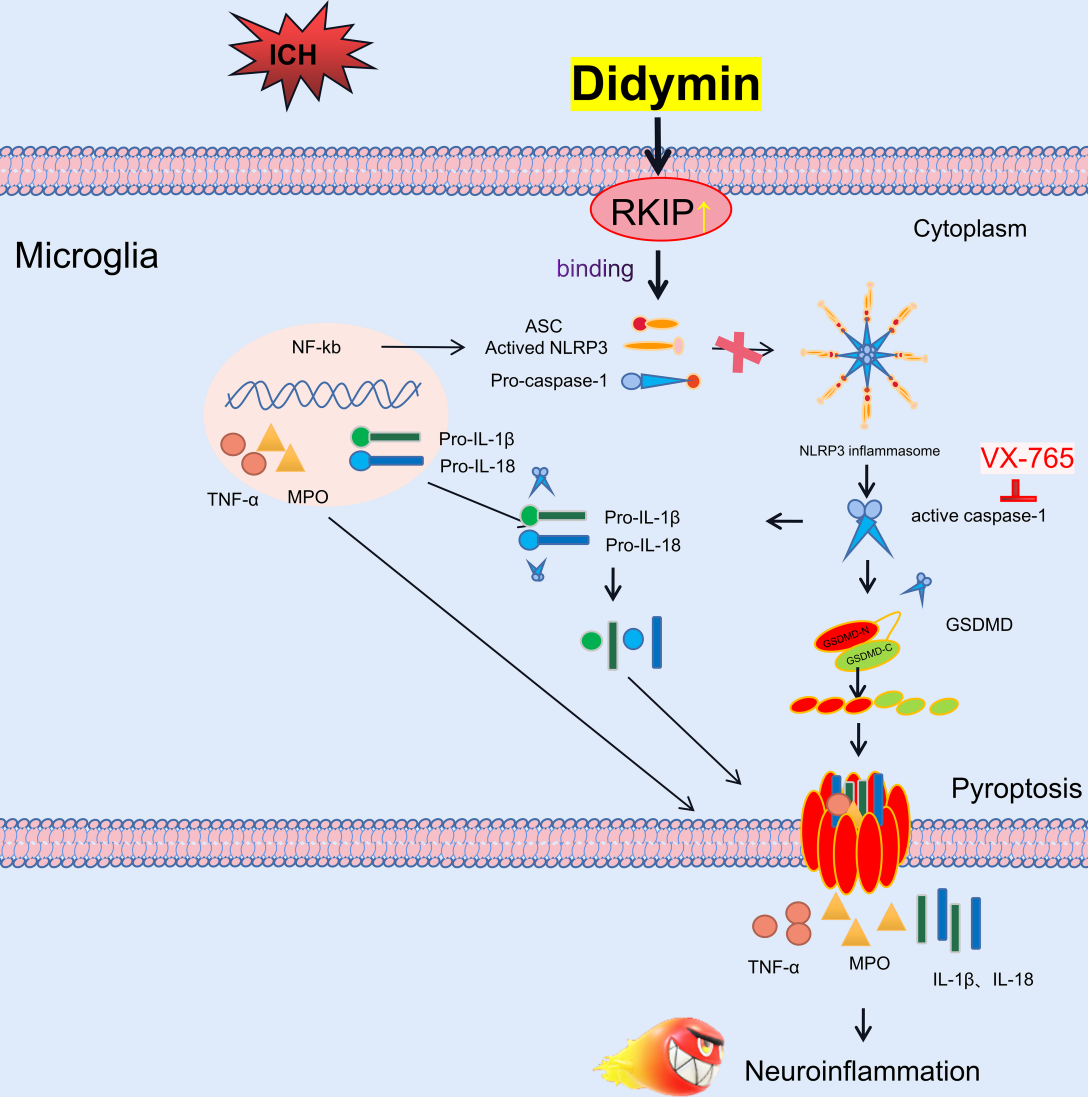
Schematic diagram of potential molecular mechanisms of the anti-neuroinflammatory effect of Didymin through the Asc/Caspase-1/GSDMD pathway following ICH.

In the present study, we first identified the effect of Didymin treatment on microglial pyroptosis and neuroinflammation following experimental ICH, which was at least in part mediated by the Asc/Caspase-1/GSDMD signaling pathway. First, administration of Didymin improved neurological deficits and attenuated brain edema and BBB disruption following ICH. Second, Didymin alleviated microglial activation and neutrophil infiltration by upregulating the expression of Rkip and downregulating the expression of pyroptotic molecules such as Nlrp3, Caspase-1, GSDMD, and inflammatory cytokines such as TNF-α and MPO after ICH. Third, inhibition of endogenous Rkip by Locostatin abolished the anti-pyroptosis and anti-inflammation effects of Didymin at 24 h post-ICH. Fourth, the Caspase-1 inhibitor ameliorated microglial pyroptosis and neuroinflammation. Finally, Rkip directly bound Asc to interrupt Nlrp3 inflammasome formation after ICH (**Figure 9**).

Mounting evidence from preclinical and clinical studies has manifested that neuroinflammation was responsible for the pathogenesis of secondary brain injury after ICH (1, 37). Neuroinflammatory response in CNS is an extremely complex process, which is just like a symphony composed by the coordinated action of different groups of glial cells (38). Activation of microglia is the initial step in the inflammatory responses, followed by infiltration of neutrophils, which amplifies the inflammatory response and renders the breakdown of the extracellular matrix, cellular integrity, and BBB (39–41). Numerous studies have demonstrated that inhibition of neuroinflammatory response could attenuate brain injury and improve neurological performance after ICH (32, 42).

Didymin, a flavonoid-glycoside compound, participates in several biological activities including anti-inflammation, antioxidant, anticancer, and antibacterial, hepatoprotective, and neuroprotective processes (23, 43–45). Moreover, a recent study suggested that Didymin treatment significantly attenuated the MPO activity and neutrophil infiltration and converted pro-inflammatory M1-like to the anti-inflammatory M2-like macrophage phenotype, thereby alleviating the clinical symptoms of colitis (25). In the present study, inflammatory response with extensive microglial activation and aberrant neutrophil infiltration in the ipsilateral cortex was induced during the acute stage of ICH. Didymin treatment evidently mitigated microglial activation and neutrophil infiltration and inhibited the release of inflammatory cytokines such as IL-1β, TNF-α, and MPO, thus attenuating neuroinflammation and brain injury after ICH.

Pyroptosis, redefined as gasdermin-mediated programmed cell death, hallmarks plasma membrane pore formation and following massive leakage of cytosolic contents, such as pro-inflammatory cytokines (19). Canonical pyroptosis initiated by the Nlrp3 inflammasome can amplify the inflammatory response by activating its core component Caspase-1, which cleaves GSDMD, pro-IL-1β, and pro-IL-18 (46–49). The N-terminal fragment of GSDMD forms pores in the plasma membrane of cells, resulting in its permeability to drainage inflammatory intracellular contents, which eventually aggravated ICH-induced neuroinflammation (50). Hence, in essence, pyroptosis is a lytic inflammatory type of programmed cell death; its occurrence leads to the release of pro-inflammatory cytokines IL-1β and IL-18 to cause inflammatory responses (51, 52). In the current study, we first observed that administration of Didymin inhibited the expression of pyroptotic molecules including Nlrp3, Caspase-1, and GSDMD, as well as the number of Caspase-1-positive microglia after ICH. These results indicated that Didymin inhibited microglial pyroptosis after ICH.

Subsequently, the underlying mechanism and signaling pathway responsible for Didymin-mediated microglial pyroptosis and neuroinflammation were explored. Rkip, a multifunctional protein, negatively regulates inflammatory response. The expression of Rkip was decreased in various cancers and neurodegenerative diseases, such as AD and PD (53–56). In the current study, the expression of endogenous Rkip was downregulated in the experimental ICH, which corresponded with the previous study in the cerebral ischemia model (57, 58). These results suggested that the downregulation of Rkip had a close bearing on the progression of brain injury after ICH. Previous studies demonstrated that Didymin exerted a protective effect in CCl4-induced hepatic injury and neuroblastoma *via* upregulating Rkip (23, 59). In our study, endogenous Rkip was decreased after ICH, while Didymin treatment upregulated Rkip expression. To gain a closer view of the correlation between the neuroprotective effects of Didymin and Rkip, Locostatin was used to inhibit endogenous Rkip expression. Our results showed that Locostatin remarkedly abolished the neuroprotective effects of Didymin on microglial pyroptosis and neuroinflammation. Furthermore, a recent study indicated that Rkip binding with Asc negatively regulated the formation and activation of inflammasome in macrophages (26). Our study revealed that Rkip directly bound with Asc by Co-IP assays. These results suggested that Didymin exerted anti-pyroptosis and anti-neuroinflammation effects *via* upregulation of Rkip expression, which could bind with Asc to interrupt Nlrp3 inflammasome formation. Canonical inflammasome depends on the Caspase-1 pathway for the maturation and secretion of IL-1β and GSDMD for the induction of pyroptosis, in which GSDMD is regarded as the direct and final executor of pyroptotic cell death (9, 60). To be specific, activated caspase-1 cleaves GSDMD, the N-terminal and C-terminal cleavage fragments, in that the N-terminal fragment of GSDMD forms pores in the plasma membrane of cells, resulting in its permeability to drainage inflammatory intracellular contents (50), and activated caspase-1 cleaves pro-IL-1β and pro-IL-18 (49). Accumulating evidence has demonstrated that inhibition of Caspase-1 exerts neuroprotective effects against BBB injury (61, 62). In the present study, our results showed that VX-765 improved neurological deficits and decreased the expression of pyroptotic molecules and inflammatory cytokines. Therefore, to some extent, these findings strengthened our hypothesis that the anti-pyroptosis effect of Didymin might be mediated at least in part through inhibiting the activation of the Caspase-1/GSDMD pathway.

There are some limitations in the current study. It has been reported that Didymin possesses numerous biological properties (63). In fact, we just focused on its anti-pyroptosis effect after ICH in this study. Therefore, further studies are required to elucidate other neuroprotective roles of Didymin and its underlying signaling mechanisms in brain injury after ICH. Additionally, a recent study reported that Didymin switches M1-like toward M2-like macrophage to ameliorate ulcerative colitis *via* fatty acid oxidation (25). Further studies are warranted to explore the polarization between M1-like and M2-like microglia under ICH condition. Finally, the long-term outcome and underlying mechanisms of Didymin need to be discussed in brain injury after ICH in the future.

## Conclusion

The present study demonstrates that Didymin alleviated microglial pyroptosis and neuroinflammation and improved neurological functions after experimental ICH, at least in part through the Asc/Caspase-1/GSDMD pathway *via* upregulating Rkip expression. Therefore, Didymin may serve as a promising therapeutic agent to attenuate neuroinflammation after ICH.

## Data Availability Statement

The raw data supporting the conclusions of this article will be made available by the authors, without undue reservation.

## Ethics Statement

The animal study was reviewed and approved by the Animal Ethics Committee of Chongqing Medical University.

## Author Contributions

LG and ZX designed the research. LG, MS, XZ, and YY performed the experiments. LG, YT, and RL analyzed the data. LG and ZX wrote the manuscript. All authors contributed to the article and approved the submitted version.

## Funding

This study was funded by the Kuanren Talents Program of the Second Affiliated Hospital of Chongqing Medical University (No. 201959), Venture & Innovation Support Program for Chongqing Overseas Returnees (No. CX2019156), Chongqing Science and Health Joint Medical Research Project (No. 2020GDRC006), and Chongqing Postgraduate Scientific Research Innovation Project (No.CYS20198).

## Conflict of Interest

The authors declare that the research was conducted in the absence of any commercial or financial relationships that could be construed as a potential conflict of interest.

## Publisher’s Note

All claims expressed in this article are solely those of the authors and do not necessarily represent those of their affiliated organizations, or those of the publisher, the editors and the reviewers. Any product that may be evaluated in this article, or claim that may be made by its manufacturer, is not guaranteed or endorsed by the publisher.

## Supplementary Material

Supplementary Figure 1 The temporal expression of Rkip after ICH.
