# Supplementary figures and images for: Didymin Suppresses Microglia Pyroptosis and Neuroinflammation Through the Asc/Caspase-1/GSDMD Pathway Following Experimental Intracerebral Hemorrhage
Source: Front Immunol. 2022 Jan 27;13:810582. doi: 10.3389/fimmu.2022.810582 (PMC8828494; doi:10.3389/fimmu.2022.810582)

Figure S1

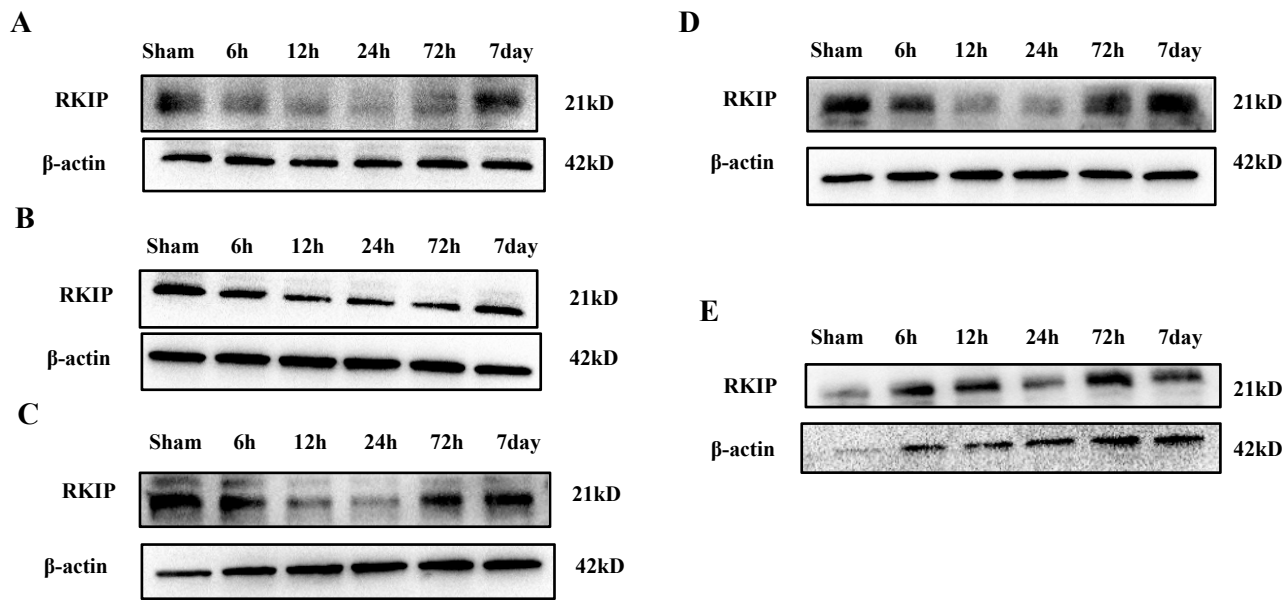

Supplement: Supplementary Figure 1 — The temporal expression of Rkip after ICH. [file Image_1.pdf]
