# Supplementary material for: Didymin Suppresses Microglia Pyroptosis and Neuroinflammation Through the Asc/Caspase-1/GSDMD Pathway Following Experimental Intracerebral Hemorrhage
Source: Front Immunol. 2022 Jan 27;13:810582. doi: 10.3389/fimmu.2022.810582 (PMC8828494; doi:10.3389/fimmu.2022.810582)
Supplement: Supplementary file 2 [file Table_1.doc]

**Table 1. Summary of experimental groups, sample sizes, and mortality rate in the study.**

| **Experimental Groups** | **Neurological test**  **Brain water content** | **Evans blue** | **IF** | **Elisa** | **WB** | **IP** | **Exclusion** | **Mortality** | | **Subtotal** | |
| --- | --- | --- | --- | --- | --- | --- | --- | --- | --- | --- | --- |
| **Experiment 1** |  |  |  |  |  |  |  |  |  | |  |
| sham | 6 | 6 |  |  |  |  | 0 | 0 | 12 | |  |
| ICH+Vehicle（DMSO）  ICH+Didymin (0.5mg/kg) | 6  6 | 6  6 |  |  |  |  | 1  1 | 2  2 | 15  15 | |  |
| ICH+Didymin (1.5mg/kg) | 6 | 6 |  |  |  |  | 1 | 1 | 14 | |  |
| ICH+Didymin (4.5mg/kg) | 6 | 6 |  |  |  |  | 1 | 2 | 15 | |  |
| **Experiment 2**  Sham |  |  |  |  | 6 |  | 0 | 0 | 6 | |  |
| ICH (6 h, 12 h, 24 h, 72 h, 7d) |  |  | 3 |  | 6×5 |  | 3 | 9 | 45 | |  |
| **Experiment 3** |  |  |  |  |  |  |  |  |  | |  |
| Sham  ICH+Vehicle（DMSO） |  |  | 3  3 | 6  6 | 6  6 |  | 0  1 | 0  2 | 15  18 | |  |
| ICH+Didymin |  |  | 3 | 6 | 6 |  | 1 | 2 | 17 | |  |
| **Experiment 4** |  |  |  |  |  |  |  |  |  | |  |
| sham | 6 | 6 | 3 | 6 | 6 |  | 0 | 0 | 27 | |  |
| ICH+Vehicle（DMSO） | 6 | 6 | 3 | 6 | 6 |  | 0 | 3 | 30 | |  |
| ICH+ Didymin | 6 | 6 | 3 | 6 | 6 |  | 0 | 1 | 28 | |  |
| ICH+Didymin+Locostatin | 6 | 6 | 3 | 6 | 6 |  | 0 | 3 | 30 | |  |
| **Experiment 5** |  |  |  |  |  |  |  |  |  | |  |
| Sham+Locostatin | 6 | 6 | 3 | 6 |  |  | 0 | 0 | 21 | |  |
| ICH+Locostatin | 6 | 6 | 3 | 6 |  |  | 0 | 3 | 24 | |  |
| ICH+Locostatin+VX-765 | 6 | 6 | 3 | 6 |  |  | 0 | 1 | 22 | |  |
| **Experiment 6** |  |  |  |  |  |  |  |  |  | |  |
| ICH (24h) |  |  | 6 |  |  | 12 | 0 | 0 | 18 | |  |
| **Total** |  |  |  |  |  |  | 8 | 30 | 372 | |  |

ICH, intracerebral hemorrhage. DMSO, Dimethyl sulfoxide.
